# Near-Infrared to T-Ray Frequency Conversion Using Kagome Photonic Crystal Resonators

**DOI:** 10.3390/nano15090663

**Published:** 2025-04-27

**Authors:** Deepika Tyagi, Vijay Laxmi, Ahsan Irshad, Abida Parveen, Mehboob Alam, Yibin Tian, Zhengbiao Ouyang

**Affiliations:** 1 THz Technology Laboratory, Shenzhen Key Laboratory of Micro-Nano Photonic Information Technology, Shenzhen University, Shenzhen 518060, China; tyagideepika33@gmail.com (D.T.); vijaylaxmigupta90@gmail.com (V.L.); aabii231@gmail.com (A.P.); 2 Key Laboratory of Optoelectronic Devices and Systems of Ministry of Education and Guangdong Province, College of Physics and Optoelectronic Engineering, Shenzhen University, Shenzhen 518060, China; 3 State Key Laboratory of Radio Frequency Heterogenous Integration, College of Mechatronics and Control Engineering, Shenzhen University, Shenzhen 518060, China; 4 Department of Physics, University of Poonch Rawalakot, Rawalakot 12350, Pakistan; ahsan.irshad.phy@gmail.com; 5 Department of Electrical Engineering, University of Poonch Rawalakot, Rawalakot 12350, Pakistan; m.alam@ieee.org

**Keywords:** Kagome lattices, photonic crystal, frequency generation, frequency conversion, resonator, beat frequency

## Abstract

Kagome lattices have attracted significant research interest due to their unique interplay of geometry, topology, and material properties. They provide deep insights into strongly correlated electron systems, novel quantum phases, and advanced material designs, making them fundamental in condensed matter physics and material engineering. This work presents an efficient method for terahertz (THz) wave generation across the entire THz spectrum, leveraging high-quality-factor Kagome-shaped silicon photonic crystal resonators. In the proposed simulation-based approach, an infrared (IR) single-frequency wave interacts with an induced resonance mode within the resonator, producing a THz beat frequency. This beat note is then converted into a standalone THz radiation (T-ray) wave using an amplitude demodulator. Simulations confirm the feasibility of our method, demonstrating that a conventional single-frequency wave can induce resonance and generate a stable beat frequency. The proposed technique is highly versatile, extending beyond THz generation to frequency conversion in electronics, optics, and acoustics, among other domains. Its high efficiency, compact design, and broad applicability offer a promising solution to challenges in THz technology. Furthermore, our findings establish a foundation for precise frequency manipulation, unlocking new possibilities in signal processing, sensing, detection, and communication systems.

## 1. Introduction

Terahertz (THz) waves, also known as T-rays, occupy the frequency range between microwaves and infrared light (0.1 to 10 THz), offering unique advantages for applications in fields such as communication, imaging, spectroscopy, and sensing [1,2,3,4,5]. However, the efficient generation of THz waves remains a significant challenge due to the lack of compact, high-power sources. Significant advancements in THz technology have been made, including innovations in THz wave generation [6], waveguides [7], spectroscopy [8], communication systems [9], optically controlled modulators [10], wave switches [11], narrowband-pass and narrowband-reject filters [12], highly efficient directional emitters [13], and biochemical sensors [14]. In this context, frequency conversion techniques play a pivotal role for generating THz radiation from more readily available sources such as near-infrared (NIR) lasers [15]. Recent frequency conversion techniques have predominantly relied on nonlinear optical processes. One widely used approach is second-harmonic generation (SHG), which employs materials such as beta barium borate and periodically poled lithium niobate to effectively double the frequency of an input wave [16]. However, SHG efficiency is highly dependent on phase-matching conditions, requiring precise material engineering and limiting its applicability across a broad frequency range. Likewise, third-harmonic generation (THG) triples the frequency of the input wave [17]. Additionally, sum-frequency generation (SFG) and difference-frequency generation (DFG) facilitate the creation of new frequencies by combining or subtracting input frequencies using nonlinear crystals and waveguides. These techniques are particularly valuable for applications in the infrared and terahertz (THz) spectral ranges [18]. However, they require high-intensity input waves and precise material engineering, leading to limited efficiency and increased implementation complexity. Another approach is the four-wave mixing (FWM) technique, which generates a new wave through the interaction of four waves in nonlinear materials [19]. A key characteristic of these methods is their reliance on the nonlinear response of the base material. Since this response depends on high-order nonlinear coefficients, achieving efficient frequency conversion typically demands extremely high input power, leading to low overall efficiency.

Linear Photonic Crystals (LPCs) are periodic dielectric structures that manipulate light through Bragg scattering rather than relying on nonlinear optical effects [20]. Their precise ability to confine and control electromagnetic waves makes them highly effective for frequency conversion and wave manipulation, positioning LPCs as a promising alternative to conventional nonlinear material-based techniques for efficient and versatile THz wave generation [21]. In LPCs, the recent developments in architectures, especially Kagome lattices, have introduced innovative strategies for THz wave generation by utilizing their superior ability to confine light and support high-Q resonance modes. Characterized by their periodic refractive index modulation, they provide unique opportunities to tailor light dispersion properties, enabling optical processes essential for efficient frequency conversion [21]. The main contributions of this paper are summarized as follows:

- Novel THz Generation Method: We propose an efficient method for generating terahertz waves across the entire THz spectrum, establishing a strong theoretical foundation and utilizing high-quality-factor Kagome-lattice photonic crystal resonators.
- Resonance-Driven Frequency Conversion: We demonstrate how an IR single-frequency wave interacts with an induced resonance mode within the resonator to produce a THz beat frequency, which is then converted into a standalone THz radiation wave (T-ray) using an amplitude demodulator.
- Versatile and Efficient Approach: Unlike conventional nonlinear optical techniques (e.g., second-harmonic generation, sum/difference frequency generation, four-wave mixing), the proposed method utilizes linear photonic crystals to achieve frequency conversion with high efficiency and compact design.
- Simulation-Based Validation: We provide numerical simulations confirming the feasibility of the approach, demonstrating stable beat frequency generation and effective resonance coupling.

These contributions position the work as a significant step forward in THz technology, addressing key challenges in efficient, compact, and high-performance THz wave generation. The following section outlines the theoretical foundation of the proposed Kagome-lattice photonic crystal resonator, followed by an in-depth discussion of its design and configurations. Section 3 presents the design analysis and simulation results, while the final section concludes the paper.

## 2. Frequency Conversion in Kagome-Lattice Photonic Crystal Resonators

Photonic crystal resonators offer a powerful platform for frequency conversion by leveraging resonance effects and wave interference. In this work, we utilize the sideband of the Lorentz-line shape or other line shapes of a source wave to excite the fundamental resonance of a Kagome-lattice photonic crystal resonator, leading to beat note generation in the THz range. Through numerical simulations and analytical modeling, we demonstrate the feasibility of this approach, highlighting its potential for efficient THz wave generation and frequency conversion.

### 2.1. Sideband Interactions and Beat Note Generation

We harness the sideband of the Lorentz-line shape of a source frequency to excite the fundamental resonance of a photonic crystal resonator. The influence of an external field on the resonator mirrors its effect on artificial atoms, driving effective charge oscillations as described by the Lorentz model [22]. Through edge effects or sideband interactions, a single-frequency wave interacting with a structured medium or resonant system can give rise to additional frequency components [22,23]. When the central peak frequency of the input wave differs from the resonator’s fundamental resonance, their interaction within the cavity generates beat notes in the THz range. To validate this phenomenon, we employ numerical simulations of Maxwell’s equations using the plane-wave expansion method [24], the finite-difference time-domain (FDTD) technique [25], and analytical modeling. This method offers a versatile framework for frequency generation and conversion, extending its applicability across diverse classical systems, including electronics, acoustics, mechanics, electromagnetism, optics, and quantum technologies.

In the proposed structure, the generated beat note $f_{B}$ corresponds to the absolute frequency difference between the source wave frequency $f_{1}$ and the cavity’s resonance frequency $f_{2}$, expressed as

(1) $$\begin{array}{c} f_{B}=\left|f_{1}-f_{2}\right|. \end{array}$$

The power-conversion efficiency (PCE), denoted as $\eta_{p}$, can be determined using the following expression:

(2) $$\begin{array}{c} \eta_{p}=\frac{E_{cf}^{2}}{E_{sf}^{2}}, \end{array}$$

where $E_{sf}$ and $E_{cf}$ are the electric field amplitudes of the source wave and the net converted frequency wave, respectively. The input wave frequency and the resonator’s resonance frequency lie within the photonic band gap (PBG), whereas the resulting beat frequency is significantly lower and falls outside the PBG. Consequently, the photonic crystal (PhC) functions as a low-pass filter. To extract the THz wave, an optical amplitude demodulator is placed at the center of the PhC resonator, converting the beat note into an independent THz signal that radiates freely from the structure.

To achieve efficient terahertz operation, Kagome-shaped PhC resonators or cavities are designed using silicon (Si) rods arranged in air, allowing interaction with source light in the infrared communication waveband. In the proposed approach, the beat note arises from the interference between the source wave and the sideband-induced resonance wave within the PhC cavity. For optimal interference, the group velocity of the source wave must be minimized. Ideally, the source wave should be confined within the cavity with zero group velocity, which is achieved by tuning the cavity resonance frequency to closely match the source wave frequency. As a result, the source wave remains nearly resonant with the cavity, exhibiting an almost zero frequency group speed [26].

### 2.2. Lorentzian Spectral Distribution and the Edge Effect

In conventional scenarios, the sidebands of a single-frequency input wave are often regarded as an insignificant or undesired component of the signal, contributing little to practical applications. However, in this work, we take a different approach by harnessing these sidebands to excite a photonic crystal resonator, thereby inducing a distinct and observable resonance mode. This resonance mode then interacts with the input wave, leading to the generation of beat notes, which are instrumental in our proposed frequency conversion mechanism. To illustrate the fundamental principles underlying this phenomenon, we draw an analogy to a mechanical oscillator consisting of a charged particle attached to the end of a spring. When subjected to an external electric field, the charged particle undergoes oscillatory motion, which can be described by the following equation of motion:

(3) $$\begin{array}{c} \frac{d^{2}r}{dt^{2}}+\beta \frac{dr}{dt}+\omega_{0}^{2}r=\frac{q}{m}E_{0}\cos\left(\omega t\right), \end{array}$$

where *r* is the displacement of the oscillating particle in the oscillator, *t* is time, *m* and *q* are the mass and charge of the particle, β = γ⁄m, γ is the damping coefficient, $E_{0}$ is the amplitude of the source electric field, $\omega_{0}$ = $\sqrt{K/m}$, and *K* is the elastic coefficient. This equation has the following steady-state solution:

(4) $$\begin{array}{c} r\left(t\right)=\frac{\frac{q}{m}}{\sqrt{{\left(\omega^{2}-\omega_{0}^{2}\right)}^{2}+{\left(\beta \omega \right)}^{2}}}E_{o}\cos\left(\omega t+\delta \right), \end{array}$$

where $\delta =atan(\beta \omega /\left(\omega^{2}-\omega_{0}^{2}\right))$. Equation (4) shows that the displacement amplitude reaches its maximum when the source field frequency is equal to the resonance frequency of the oscillator system. However, the oscillation frequency of the particle is strictly determined by the frequency of the applied electric field, meaning that the oscillator itself does not inherently generate any new frequencies beyond that of the source wave. In this scenario, the driving electric field is assumed to be a perfectly monochromatic, single-frequency wave with an idealized zero bandwidth.

In practical scenarios, however, no real-world source can be considered a truly ideal single-frequency wave. Instead, all single-frequency sources exhibit a spectral profile with a finite bandwidth. A common example is the Lorentzian spectral distribution, which characterizes the frequency spread of many resonant systems. This spectral profile accounts for the natural linewidth of the source, which arises due to inherent physical limitations such as damping, phase noise, and energy dissipation. The Lorentzian spectral shape can be mathematically expressed as follows:

(5) $$\begin{array}{c} L\left(\omega \right)=\frac{1}{\pi }\frac{\Gamma /2}{{\left(\omega /\omega_{c}-1\right)}^{2}+{\left(\Gamma /2\right)}^{2}}. \end{array}$$

Here, $\omega_{c}$ represents the central frequency of the Lorentzian spectral profile, while Γ defines the full-width at half-maximum (FWHM), characterizing the spectral bandwidth. This implies that a so-called single-frequency wave is, in reality, a broadband signal encompassing a range of frequencies.

The Lorentzian function does not vanish at frequencies far from $\omega_{c}$; instead, it has long tails, meaning that there are always small but nonzero spectral components at frequencies away from $\omega_{c}$. The term “edge effect” refers to the fact that the energy distribution extends beyond $\omega_{c}$, particularly at the edges of the spectrum where the amplitude is still nonzero. This means that even though we refer to the wave as “single-frequency”, in reality, it consists of multiple frequency components. These sideband components can interact with a resonant system, leading to frequency mixing, beat notes, and new observable resonances. The Lorentzian function, which attains a peak value of $L\left(\omega_{c}\right)=2/\left(\pi \Gamma \right)$, can be normalized, so that its integral over all frequencies sums to unity:

(6) $$\begin{array}{c} \int_{-\infty }^{\infty }L\left(\omega \right)=1\;. \end{array}$$

Therefore, when considering the edge effect of the Lorentz-line shape of a practical driving source, (3) is modified as follows:

(7) $$\begin{array}{c} \frac{d^{2}r}{dt^{2}}+\beta \frac{dr}{dt}+\omega_{0}^{2}r=\frac{qE_{0}}{m}\int_{-\infty }^{\infty }\frac{L(\omega )}{B_{\omega }}\cos\left(\omega t\right)d\omega , \end{array}$$

where $E_{0}$ is a constant specifying the amplitude of the total field, and $B_{\omega }$ is a normalization factor ensuring the correct amplitude scaling of the spectral distribution. Equation (7) reveals that a nominally single-frequency source can, in reality, induce oscillations at an infinite number of additional frequencies due to the edge effect associated with the Lorentzian spectral shape of the source. This effect arises because practical single-frequency waves exhibit a finite spectral width rather than being perfectly monochromatic. In general, the Lorentzian spectrum is sharply peaked around its central frequency and decays rapidly away from it, making these additional frequency components difficult to observe. However, when the oscillator’s resonance frequency coincides with a significant spectral component of the source, the resulting motion at that frequency can become pronounced. This effect is particularly noticeable when the oscillator or resonator has a high-quality (*Q*) factor, allowing it to sustain and amplify resonant oscillations, and when the coupling between the source and the oscillator is strong.

Since the resonance component is significantly stronger than other frequency components, except for the fundamental source frequency, Equation (7) can be approximated as follows. For simplicity, we assume that the oscillator supports only a single dominant resonance mode:

(8) $$\begin{array}{c} \frac{d^{2}r}{dt^{2}}+\beta \frac{dr}{dt}+\omega_{0}^{2}r\approx \frac{qE_{0}}{m}\left[L\left(\omega_{0}\right)\cos\left(\omega_{0}t\right)+L\left(\omega_{c}\right)\cos\left(\omega_{c}t\right)\right]. \end{array}$$

Equation (8) clearly illustrates that under the excitation of a realistic single-frequency field, a new frequency component emerges, corresponding precisely to the resonance frequency of the oscillator. This forms the fundamental principle of resonance-induced mode generation, which plays a crucial role in this work as one of the contributing waves for producing the beat note. It is important to note that the two terms on the right-hand side of Equation (8) represent the dominant Fourier components of the integral expression in Equation (7). In particular, the second term in Equation (8), which arises from the sideband of the Lorentzian spectrum, acts as an additional driving term for the oscillator. This contrasts with a damping term, which typically manifests as an exponential decay factor in the system’s response. From Equation (4), it can be observed that when the source frequency is close to the resonance frequency, the oscillator exhibits a strong response due to resonance amplification. Moreover, the quality factor Q of the resonator is inversely proportional to the damping coefficient β. Thus, a high-Q resonator can sustain an observable response even when the source frequency is significantly detuned from the resonance frequency. This suggests that in systems with a high-quality factor, an externally applied field with a frequency far from the resonator’s natural frequency can still excite a detectable resonance-induced frequency component, but conversion efficiency decreases.

As is well known, an amplitude demodulator extracts the envelope of a modulated wave. In this context, the THz beat note, arising from the interference of two infrared waves with slightly different frequencies, can be converted into a standalone THz wave using an amplitude demodulator. Notably, the input source wave and the cavity’s resonance modes coexist within the same spatial region, enabling effective interference and beat note generation [27]. The electric fields of the two interfering waves can be expressed as follows:

(9) $$E_{j}\left(x,y,z,t\right)=E_{j}e_{j}\left(y,z\right)\cos\left(k_{j}x-\omega_{j}t\right),$$

where j=1,2 correspond to the source and resonance mode, respectively. Here, $E_{j}$ denotes the amplitude of the source and resonance mode, $e_{j}\left(y,z\right)$ represents the transverse field profile, and $k_{j}$ is the propagation constant. The coordinate *x* indicates the direction of the wave transmission. The two waves together in the cavity, and thus the total field in the cavity, can be expressed as follows:

(10) $$E_{bt}=\sum_{j=1}^{2}E_{j}\left(x,y,z,t\right).$$

Without loss of generality, we assume $E_{1}=E_{2}=E_{0}$—i.e., the source and the induced resonance fields have equal amplitudes. Furthermore, based on the same symmetry, we can assume $e_{1}\left(y,z\right)=e_{2}\left(y,z\right)=e\left(y,z\right)$. Therefore, we obtain

(11) $$E_{bt}=2E_{o}e\left(y,z\right)\cos\left(\frac{\Delta k}{2}x-\frac{\Delta \omega }{2}t\right)\cos\left(k_{3}x-\omega_{3}t\right),$$

where

(12) $$\Delta k=k_{1}-k_{2},$$

(13) $$\Delta \omega =\omega_{1}-\omega_{2},$$

(14) $$k_{3}=\frac{\left(k_{1}+k_{2}\right)}{2},$$

(15) $$\omega_{3}=\frac{\left(\omega_{1}+\omega_{2}\right)}{2}.$$

Since the two waves coexist spatially and interfere within the same region, we can express the resulting field as follows:

(16) $$k_{1}=\frac{n\omega_{1}}{c},$$

(17) $$k_{2}=\frac{n\omega_{2}}{c},$$

(18) $$\Delta k=\frac{n(\omega_{1}-\omega 2)}{c},$$

where *n* is the effective refractive index of the region where the beating occurs, and *c* is the speed of light in free space. Considering the general case where $\omega_{1}\approx \omega_{2}$, the slowly varying amplitude of the resulting beat wave can be expressed as

(19) $$E_{3}=2E_{o}e\left(y,z\right)|\cos\left(\frac{\Delta k}{2}x-\frac{\Delta \omega }{2}t\right)|>0.$$

This leads to the following expression:

(20) $$E_{bt}=E_{3}\cos\left(x-\omega_{3}t+\phi \right),$$

where

(21) $$\varphi =\left\{\begin{array}{cc} 0 & \mathrm{for}\;\cos\left(\frac{\Delta k}{2}x-\frac{\Delta \omega }{2}t\right)\geq 0,\\ \pi & \mathrm{for}\;\cos\left(\frac{\Delta k}{2}x-\frac{\Delta \omega }{2}t\right)<0. \end{array}\right.$$

From Equations (19) and (20), it is evident that the frequency of the beat note is Δω, not Δω/2. This is because the absolute value of the cosine function effectively doubles the frequency component present in the cosine term describing the amplitude of the beating wave in Equation (19). Consequently, an amplitude demodulator can be employed to extract the beat note and generate an independent wave with a frequency equal to the difference between the two input source waves. Based on Equation (19), we can write the following:

(22) $$E_{3}=2E_{o}e\left(y,z\right)\sum_{j=0}^{\infty }p_{j}\cos\left(j\frac{\Delta k}{2}x-j\frac{\Delta \omega }{2}t\right),$$

where $p_{j}$ is the series coefficient.

This phenomenon has important implications for novel applications in terahertz wave generation, an area that has remained largely unexplored. Traditionally, the spectral bandwidth of a laser wave or the sidebands of a practical sinusoidal wave have been considered negligible or without practical use. However, our analysis suggests that these sidebands can serve as effective excitation sources for frequency conversion. While the above discussion assumes that the spectral profile of a practical single-frequency source follows a Lorentzian distribution, the principle remains valid for other spectral shapes, such as Gaussian profiles. This is because all such spectral line shapes inherently exhibit sideband components, which can similarly contribute to frequency generation, leading to equations analogous to Equation (8).

### 2.3. Design and Configuration of Kagome-Lattice Photonic Crystal Resonator

**Figure 1:**
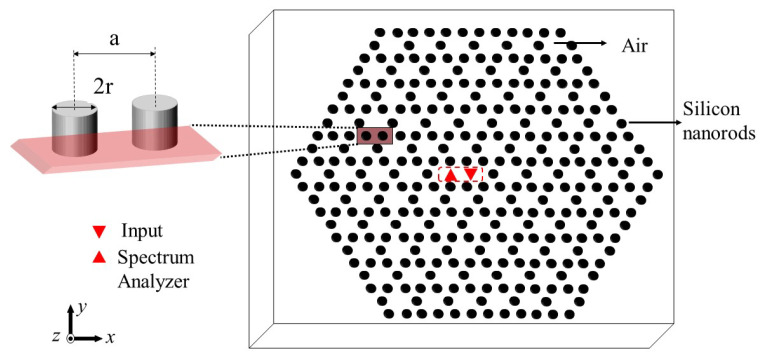
Schematic of a cavity in a Kagome-lattice PhC. White dots represent Si cylinders embedded in an air background (pink), with a lattice period of 0.6 μm. The central triangle denotes an IR source, while a monitor (IR amplitude demodulator) is positioned at the cavity’s center to detect the THz beat note or convert it into a T-ray. Schematic of a cavity in a Kagome-lattice PhC. White dots represent Si cylinders embedded in an air background (pink), with a lattice period of 0.6 μm. The cavity is formed by removing a single Si nanorod at the center of the structure, which is highlighted with a red dashed outline. The central triangle denotes an IR source, while a monitor (IR amplitude demodulator) is positioned at the cavity’s center to detect the THz beat note or convert it into a T-ray. The red markers indicate the input source and spectrum analyzer. The coordinate system is illustrated in the schematic.

Frequency conversion in a Kagome-lattice photonic crystal resonator is achieved through the interaction between the input source wave and the cavity resonance, generating a new frequency. In this section, the design and configuration of the Kagome-Lattice photonic crystal resonator in Figure 1 are discussed. It is important to observe here that by optimizing the PhC structure and cavity parameters, the source wave is strongly confined within the PBG, enabling efficient frequency mixing. The resulting beat frequency is extracted as a new THz wave, demonstrating the potential of PhC-based resonators for advanced optical signal processing. This work focuses on optical frequency conversion using a cavity resonator, designed within a two-dimensional (2D) Kagome-lattice photonic crystal. The PhC is formed by periodically arranging silicon (Si) rods (refractive index 3.4) in an air background with a lattice constant of 0.6 μm. High-quality factors are ensured by incorporating more than five reflection rows of unit cells in the resonators. Optimal coupling is achieved by positioning the source at the center of the resonators.

**Figure 2:**
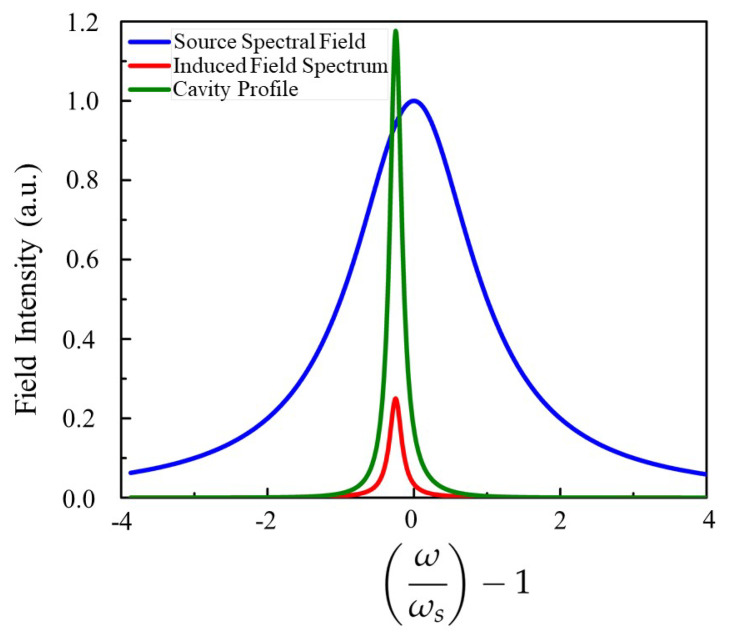
Induced field (red) in a cavity from an external source field (blue). The green curve indicates the intrinsic resonance spectrum of the cavity.

The designed structure of the Kagome-Lattice photonic crystal resonator for the generation of new frequency is shown in Figure 1, where the resonator cavity consists of a single cavity. The cavity in the resonance mode was identified near a wavelength within the PhC, so in the PBG, the waves are predominately confined inside the cavity. In the design, the source is a TE-polarized wave operating at 1.5 μm within the infrared communication band. The cavity exhibits natural resonance modes, which are intentionally designed to differ from the input source wave’s frequency. To determine these resonance modes, simulations are conducted using an impulse source and analyzed through FFT within the FullWAVE module of RSoft, (Synopsys Inc., Sunnyvale, CA, USA), employing the finite-difference time-domain (FDTD) method for accurate results. Once the resonance characteristics of the structure are determined, the identified resonance mode is utilized as one of the key components in generating the beating phenomenon. Within the cavity, the interaction between the input source wave and the resonance mode leads to the formation of a THz beating wave. This beating wave can then be processed and converted into an independent THz wave (T-ray) using an infrared (IR) amplitude demodulator. It is important to explain the phenomenon of self-induced beating. Assume, without loss of generality, that the external field has a central frequency $\omega_{s}$, slightly detuned from the cavity’s intrinsic resonant frequency. This is illustrated in Figure 2, where the blue curve represents the spectral profile of the external field (centered at $\omega_{s}$), and the red curve corresponds to the cavity spectrum. When the source field is coupled into the cavity, an induced field (red curve in Figure 2) is generated. The interference between the source and induced fields produces a beat note. For the induced field’s intensity to be comparable to the source field, the central frequencies of both spectra must be very close. This small frequency difference is key to the proposed method, as it allows an infrared source and a suitably tuned infrared-wave cavity to generate a THz beat frequency, where the required THz frequency corresponds to the slight difference between the external and intrinsic frequencies. In order to further justify the function of the PhC cavity, it is important to observe that the cavity facilitates beat frequency generation through spectral overlap between the input source wave and the cavity mode. When two slightly detuned waves interact—one following the source field spectrum (blue curve, Figure 2) and the other aligning with the cavity resonance (red curve, Figure 2)—their interference produces a modulated intensity pattern, generating a THz beat frequency. The cavity enhances this process by confining the optical field, ensuring efficient interaction. The optical amplitude demodulator then extracts the THz signal by detecting intensity modulation, demonstrating the viability of PhC-based frequency conversion for advanced optical signal processing.

Establishing a clear connection with the mechanical oscillator analogy discussed in Section 2.1, particularly the transition from Equation (7) to Equation (8), requires emphasizing that the source field spectrum is not purely monochromatic but instead exhibits a finite bandwidth, as illustrated in Figure 2. The Lorentzian function describing the source field does not vanish at frequencies away from its central frequency, leading to small but nonzero spectral components across a range of frequencies. These sideband components can interact with the resonator, giving rise to additional frequency contributions beyond the fundamental mode. In Equation (8), we approximate the system response by selecting the dominant frequency components from Equation (7), justified by the fact that a high-Q resonator can sustain a measurable response even for source frequencies detuned from its natural resonance [28]. This aligns with the physical behavior observed in Figure 2, where both the source field and cavity profile exhibit finite spectral widths, reinforcing the necessity of considering a broader frequency distribution rather than assuming a strictly monochromatic excitation. The subsequent analysis demonstrates that, with appropriately chosen parameters, T-rays can be effectively generated from near-infrared (NIR) waves, highlighting the feasibility and practical application of this frequency conversion approach.

For effective wave confinement within the cavity, the wave frequency must fall within the PBG of the PhC. A wider bandgap enhances confinement, improving the overall performance. To optimize this, the plane-wave expansion method was utilized alongside the BandSOLVE module in RSoft to scan the PhC parameters. This analysis revealed a relatively large bandgap for the TE wave in the Kagome-lattice PhC, achieved at a rod radius of R=0.2a, where *a* denotes the lattice constant. A normalized frequency of a/λ=0.325 was selected for the operation. To demonstrate the validity and general applicability of the method, the source frequency was not positioned at the center of the bandgap. However, for practical applications, selecting a source frequency at the middle of the PBG can enhance the cavity’s quality factor, leading to stronger wave confinement. To further analyze the performance, simulations were conducted using the Fullwave FDTD module in RSoft for the structure illustrated in Figure 1. The impulse mode and continuous wave (CW) mode in RSoft are utilized to simulate and analyze resonance characteristics and the evolution of the electric field, respectively.

**Figure 3:**
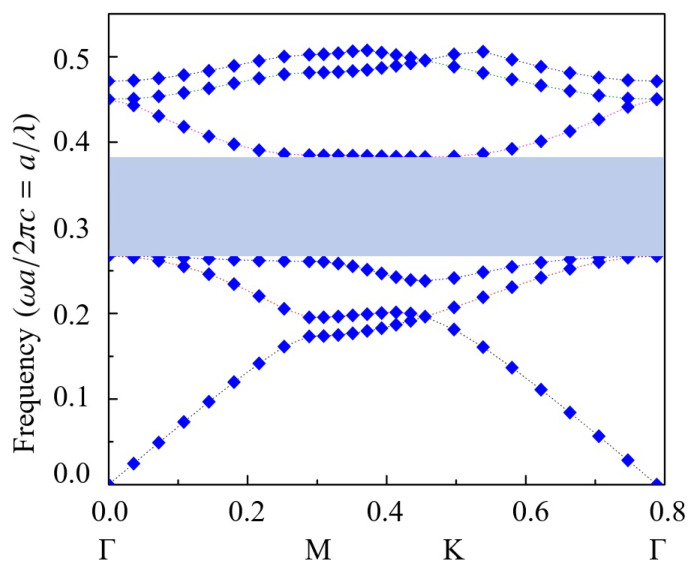
Band structure of a 2D Kagome-lattice photonic crystal composed of Si cylinders in air, with a cylinder radius of R=0.2a, where *a* is the lattice constant. The plot illustrates a relatively wide bandgap at lower frequencies and a narrower bandgap at mid-range frequencies for TE waves.

The bandgap of the PhC structure can be tailored through design modifications, enabling precise control over light propagation and manipulation within the material. Additionally, reducing or eliminating TM bandgaps enhances the confinement and control of TE-polarized light in targeted regions, improving efficiency, signal quality, and overall device performance. The gap-to-mid-gap ratio (GMR), which quantifies the bandgap size, is determined to be 0.375 from Figure 3. However, an optimal GMR value does not exist universally, as it varies based on the specific application, balancing performance requirements, fabrication complexity, and material constraints. In practical applications, the following empirical guidelines are commonly used to determine suitable GMR values:

- 0.1 < GMR ≤ 0.2: Sufficient for most low-index contrast systems and simple devices.
- 0.2 < GMR ≤ 0.3: Ideal for many integrated photonic devices (waveguides, resonators).
- GMR > 0.3: Optimal for advanced applications requiring high confinement or broadband operation.

In the following section, simulations of the designed Kagome-lattice photonic crystal resonator configuration are presented. The FullWAVE module in RSoft is utilized to obtain results for the structure shown in Figure 1. The simulation setup includes perfectly matched layers to prevent boundary reflections, while small time steps and finely tuned geometrical grid sizes are implemented to ensure stability and high precision. A detailed discussion of the simulation process and results follows in the next section.

## 3. Results and Discussion

This section discusses the proposed creation of new frequencies using a single-cavity resonator within a Kagome-lattice PhC. A localized resonance mode is achieved by removing a single Si rod from the periodic arrangement, facilitating controlled optical frequency conversion. The interaction between the input wave and the cavity resonance results in a THz beat frequency, which can be harnessed for various practical applications.

### 3.1. Frequency Generation Using Single-Cavity Structure in Kagome-Lattice Photonic Crystal Resonator

**Figure 4:**
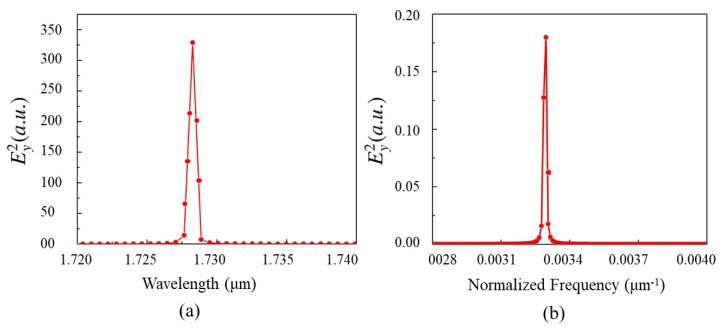
FFT response of the electric-field intensity ($E_{y}^{2}$) for (**a**) the single-cavity resonator in impulse mode to find the resonance of the structure, showing a resonance peak at 1.7283 μm with an FWHM of 0.0006 μm, and (**b**) the single-cavity resonator in continuous wave mode revealing a beating wave at the normalized frequency of f/c= 0.0033 μm^−1^ or 1 THz with an FWHM of 0.0017 μm. The CW source spectrum is generated via Fourier transform of a Gaussian time-domain pulse.

This section presents the generation of new frequencies using a single-cavity resonator in a Kagome-lattice PhC. The structure consists of Si rods, each with a radius equal to 0.2 times the lattice constant, periodically arranged in air with a lattice period of 0.6 μm. To create a localized resonance mode, a single Si rod is removed from the center of the structure, forming a single-cavity resonator, as shown in Figure 1. A monitor was positioned at the center of the cavity to capture the resulting wave interactions. In the schematic, the white region represents air, while the black circles denote dielectric poles. The monitor size was set to 0.5a, where *a* is the lattice constant, ensuring minimal edge effects and accurate simulation results. The source was placed near the center of the cavity. Initially, a resonance mode was identified using a broad-band impulse source with its central wavelength at 1.5 μm and Fast Fourier Transform (FFT) analysis, as illustrated in Figure 4a.

To generate the desired beating frequency, the source frequency was tuned slightly offset from the resonator’s resonance frequency by an amount corresponding to the target beating frequency. As a result, a strong resonance mode was obtained at 1.7283 μm for the single-cavity structure using an impulse source, with a high-quality factor of 2880 as shown in Figure 4a. The quality factor (*Q*) is defined as

(23) $$\begin{array}{c} Q\;\;=\;\;\frac{\mathrm{Resonance}\;\mathrm{frequency}\;\omega_{0}}{\mathrm{Bandwidth}\;\Delta \omega } \end{array}$$

$\omega_{0}$ is the central resonant frequency and Δω is the full-width at half-maximum (FWHM) bandwidth of the resonance peak in the frequency domain. This exceptionally high *Q*-factor in PhC designs indicates superior light confinement and minimal energy loss, which are primarily influenced by two key design parameters. The first is the number of unit cell rows surrounding the cavity. To achieve a high *Q*-factor, at least five rows of unit cells are typically required around the cavity center. The second is the choice of low-loss materials for the dielectric rods. In the infrared band, silicon exhibits minimal loss, making it an ideal material for maximizing light confinement and enhancing the overall performance.

The beating frequency in the THz range is achieved by selecting the resonance peak wavelength and adjusting the source wavelength with a difference corresponding to the desired THz frequency. In the simulation, the resonance peak at 1.7283 μm corresponds to a frequency of 173.46 THz. Setting the source in CW mode at 1.7183 μm (174.46 THz) results in a beating frequency of 1 THz. The simulation results clearly demonstrate the generation of a new frequency, which interacts with the source wave to produce a beat wave with a beating frequency of 1 THz, or f/c=0.0033 μm^−1^, as shown in Figure 4b. Multiple resonance frequencies can exist depending on the number of cavities in the structure, while the period and dielectric rod radius have been optimized for the widest bandgap. When multiple resonance frequencies are present, any of them can be used to generate beating waves, with preference given to those within the bandgap and exhibiting a high quality factor.

**Figure 5:**
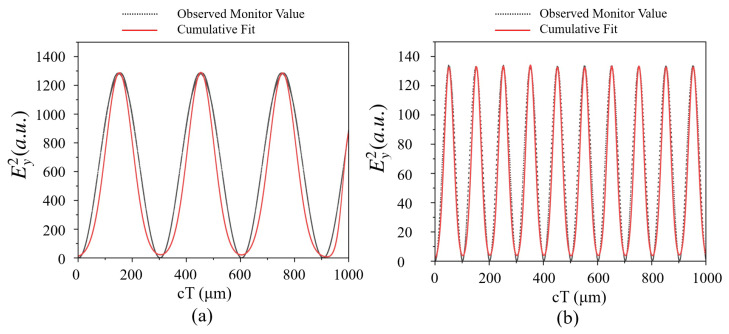
Electric-field intensity ($E_{y}^{2}$) envelope of the modulated wave for a single-cavity resonator with a single source, evaluated at the cavity center. The results correspond to the operating source wavelengths of (**a**) 1.7183 μm and (**b**) 1.6989 μm in the Kagome-lattice PhC, generating beating frequencies of 1 THz and 3 THz, respectively.

**Figure 6:**
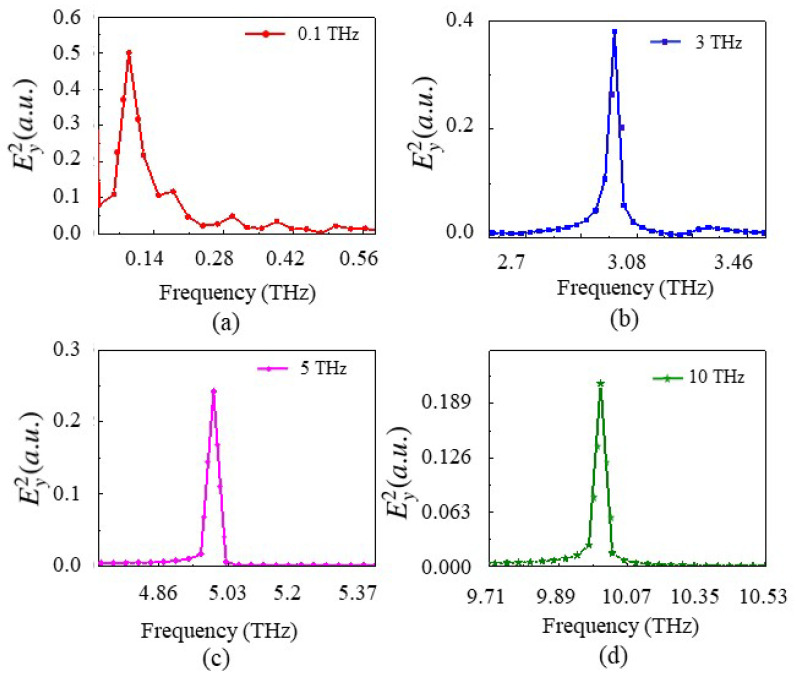
FFT response of electric-field intensity ($E_{y}^{2}$) for single-cavity resonator in continuous wave mode to generate (**a**) 0.1 THz, (**b**) 3 THz, (**c**) 5 THz, and (**d**) 10 THz.

Similarly, beating frequencies of 0.1 THz, 3 THz, 5 THz, and 10 THz were obtained for source wavelengths of 1.72 μm, 1.69 μm, 1.67 μm, and 1.63 μm, respectively. The electric-field intensity ($E^{2}y$) envelope of the modulated wave with respect to the normalized time for a single-cavity resonator with a single source was evaluated at the cavity center. In Figure 5, cT represents the normalized time of evolution of waves in the cavity, where c is the velocity of light (μm/s) and T is the time of evolution of an electromagnetic wave. The results correspond to the operating source wavelengths of Figure 5a, 1.7183 μm, and Figure 5b, 1.6989 μm, in the Kagome-lattice PhC, generating beating frequencies of 1 THz and 3 THz, respectively. These profiles confirm that the generated frequencies deviate slightly from the source frequency, as expected. Figure 6 illustrates the beating frequencies of 0.1 THz, 3 THz, 5 THz, and 10 THz for their respective source wavelengths. While additional frequencies can be generated using this method, frequencies above 10 THz exhibit increased noise levels, resulting in reduced signal quality. Using Equation (2), the power conversion efficiencies for 0.1 THz, 1 THz, 3 THz, 5 THz, and 10 THz are calculated as 99.25%, 95%, 92.5%, and 91.1%, respectively. Lower-frequency THz waves correspond to source frequencies that are closer to the resonance frequency. In this case, the sideband intensity of the source wave, which contributes to the generation of the THz beat note, has a higher amplitude. As a result, the output power is greater, leading to a higher power conversion efficiency. Achieving a strong beat note requires the intensity of the source wave’s central frequency component to be comparable to the resonance wave’s intensity induced in the resonator by the source wave’s sideband. This condition can be optimized by positioning the source frequency slightly away from the resonator’s resonance frequency.

For higher-frequency THz wave generation, the source frequency is significantly farther from the resonance frequency. As a result, the sideband intensity of the source wave, which contributes to generating the THz beat note, has a lower amplitude. This leads to reduced output power and lower power conversion efficiency. Consequently, the signal-to-noise ratio (SNR) for high-frequency THz waves is lower than that for low-frequency THz waves, as the absolute noise level in the system remains constant for a given environment. The beating frequencies correspond to the difference between the resonator’s resonance frequency and the source frequency, validating the proposed frequency conversion mechanism. Notably, the generated resonance frequency is independent of the source position and the number of sources. Furthermore, the generated beat signal can be converted into THz radiation using standard amplitude demodulation techniques.

### 3.2. Time Evolution of the Electric Field Distribution

**Figure 7:**
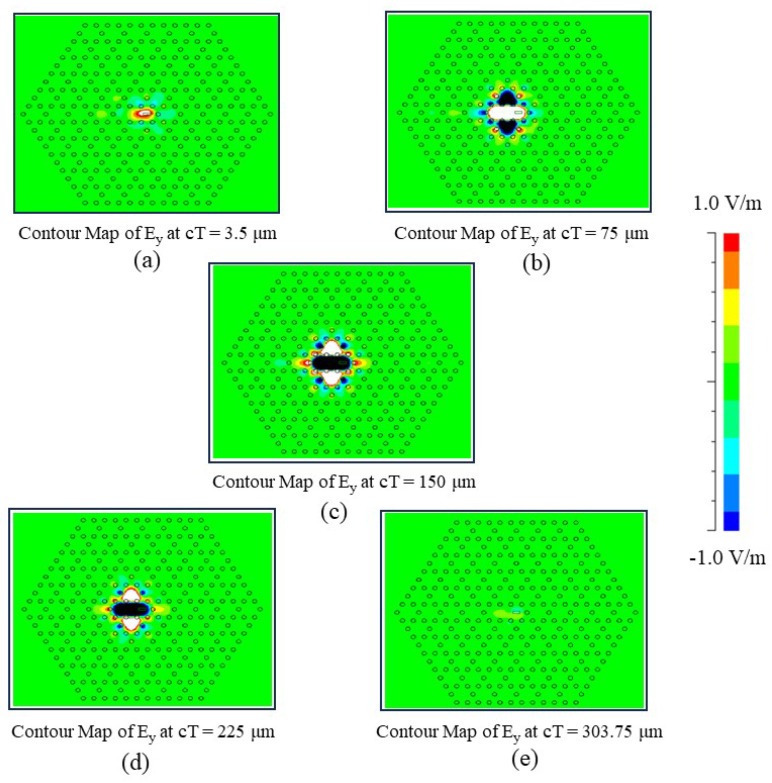
Electric field evolution versus cT value (normalized time) for one complete oscillation period of the beat note at the centre of the cavity.

The mechanism of frequency conversion in the structure is illustrated through the time evolution of the electric field distribution. Figure 7 presents the field evolution over one period of the beat note. Initially, the field in the cavity reaches its minimum at cT = 3.5 μm, at an increased value at cT = 75 μm and 150 μm, as shown in Figure 7a–c. Following this, the field amplitude begins to decrease at at cT = 225 μm and reaches its lowest value again at cT = 303.75 μm, completing one full oscillation cycle, as depicted in Figure 7d,e. The field map at cT = 303.75 μm represents the occurrence of a 1 THz wave.

The field distribution clearly demonstrates that energy initially accumulates in the central cavity before expanding into the surrounding regions. Subsequently, waves in the outer regions propagate back toward the center. Due to phase differences, the returning waves, the initial field in the central cavity, and the source wave interfere, leading to a reduction in field amplitude and forming a valley at a specific moment. The process then repeats, with the source wave regaining dominance, generating cyclic field-amplitude oscillations that produce low-frequency waves within the structure. Fabrication imperfections and variations in silicon rod dimensions can significantly impact the resonance characteristics. These imperfections may shift the resonance wavelength, causing mode splitting, or introducing distortions. The *Q*-factor, which determines the efficiency of light confinement, is highly sensitive to structural accuracy. Misalignment, surface roughness, or inconsistencies in rod size can increase scattering losses, reducing the Q-factor. Addressing these challenges requires robust design strategies, precision fabrication techniques, and tolerance-aware optimizations. By incorporating error margins into the design process, the robustness of the structure can be improved. While the effects of fabrication imperfections are design-dependent, advanced simulations and high-precision manufacturing methods can effectively mitigate these issues, ensuring stable performance in Kagome-lattice PhC resonators.

This work primarily aims to provide an efficient approach for generating THz waves, which serves as the foundation for a wide range of THz applications, including THz imaging—a challenge that remains at the forefront of modern engineering. Given its effectiveness, the proposed method holds significant potential for various practical implementations. For extending this approach to THz wave generation using an array of resonators, an array configuration can be beneficial when the input IR source has a large beam diameter. However, in most cases, IR lasers typically have a small beam diameter, making a single resonator a more efficient choice for converting IR waves into THz waves with higher efficiency.

## 4. Conclusions

In this work, we demonstrated an efficient method for THz wave generation using high-quality-factor Kagome-shaped silicon photonic crystal resonators. By leveraging the interaction between an IR single-frequency wave and an induced resonance mode, we achieved a stable THz beat frequency, which can be converted into a standalone THz radiation wave with an amplitude demodulation. Numerical simulations validated the feasibility and efficiency of our approach, highlighting its potential for compact and versatile THz generation. Beyond THz applications, our findings offer a broader framework for frequency conversion across various domains, including electronics, optics, and acoustics. This work lays the foundation for precise frequency manipulation, opening new avenues for advancements in signal processing, sensing, detection, and next-generation communication systems.

## Data Availability

The original contributions presented in this study are included in the article. Further inquiries can be directed to the corresponding authors.

## Abbreviations

The following abbreviations are used in this manuscript:

T-rays	Terahertz Radiation
THG	Third-Harmonic Generation
SFG	Sum-Frequency Generation
DFG	Difference-Frequency Generation
FWm	Four-Wave Mixing
NIR	Near Infrared
LPCs	Linear Photonic Crystals
FDTD	Finite-Domain Time-Difference
PCE	Power Conversion Efficiency
PBG	Photonic Band Gap
PhC	Photonic Crystals
Si	Silicon
FWHM	Full Width at Half Maximum
2D	Two-Dimensional
CW	Continuous Wave
TE	Transverse Electric
GMR	Gap to Mid-Gap Ratio
FFT	Fast Fourier Transform
cT	Wavelength
